# Age-Related Differences in the Limited Range of Motion of the Lower Extremity and Their Relation to Low Back Pain in Young Baseball Players: A Cross-Sectional Study of 1215 Players

**DOI:** 10.1186/s40798-023-00572-w

**Published:** 2023-05-03

**Authors:** Kinshi Kato, Kenichi Otoshi, Ryoji Tominaga, Takahiro Kaga, Takahiro Igari, Ryohei Sato, Yota Kaneko, Shin-ichi Konno

**Affiliations:** 1 Department of Orthopedic Surgery, Fukushima Medical University School of Medicine, 1 Hikarigaoka, Fukushima City, Fukushima 960-1295 Japan; 2 Department of Sports Medicine, Fukushima Medical University School of Medicine, Koriyama City, Japan

**Keywords:** Low back pain, Range of motion, Knee, Hip, Baseball, Adolescent, Age group

## Abstract

**Background:**

Age-related differences in the limited range of motion of the lower extremities and their relationship with low back pain in juvenile athletes have not been well assessed. This study investigated the relationship between low back pain and limited range of motion of the hip and knee in young baseball players during the baseball season.

**Results:**

Participants comprised 1215 baseball players (216 pitchers, 999 fielders) aged 6–16 years who underwent medical checkups (self-completed questionnaire and physical examination). Of the 1215 players, 255 (21.0%) experienced seasonal low back pain requiring rest during the previous year. The prevalence of low back pain and a positive Thomas test, straight-leg-raising test, and heel-to-buttock test increased with age. Univariate analysis revealed that a positive heel-to-buttock test in both the throwing and non-throwing arm sides in the 11–12 age group and a positive Thomas test in the throwing arm side in the 13–14 age group were associated with seasonal low back pain (*P* = 0.0051, *P* = 0.021, and *P* = 0.048, respectively). Multivariate analysis, adjusted for factors associated with low back pain, showed significant associations between the positive heel-to-buttock test (odds ratio 1.75, 95% confidence interval 1.11–2.79; *P* = 0.016) and low back pain in players aged 11–14 years.

**Conclusions:**

A positive heel-to-buttock test is potentially associated with low back pain among juvenile baseball players. Particular attention should be paid to the limited range of motion of the knee joint and tightness of the quadriceps femoris muscle among baseball players with low back pain aged 11–14 years.

**Supplementary Information:**

The online version contains supplementary material available at 10.1186/s40798-023-00572-w.

## Key Points

- Low back pain is among the most common complaints in school-aged baseball players.
- The prevalence of low back pain and a positive Thomas test, straight-leg-raising test, and heel-to-buttock test increased with age in baseball players aged 6–16 years.
- The positive heel-to-buttock test was associated with low back pain in baseball players aged 11–14 years.

## Background

Low back pain (LBP) is a universal health problem in the general population [1]. LBP is also common among competitive athletes, with an estimated prevalence range between 1 and 30% [2]. Baseball is a prominent sport in which millions of young athletes worldwide participate [3]. There is extensive literature on shoulder and elbow problems in baseball players [4]. Nevertheless, LBP, which is commonly experienced by baseball players, has been neglected [5, 6]. Baseball was the only sport identified to substantially increase the incidence of lumbar spondylolysis in a retrospective case series of 1025 non-elite adolescent athletes with LBP [7]. Another epidemiological study compared well-trained university athletes (baseball players, basketball players, kendo competitors, runners, soccer players, and swimmers) to non-athlete university students and found that continuous competitive baseball and swimming activities during youth may be associated with disk degeneration [8]. Chronic LBP affects 1–40% of baseball players with all experience levels [9, 10]. Thus, baseball may be considered a sport with a higher risk of LBP [7–10], which might be a severe obstacle to continuing baseball activities for baseball players [7–9].

Limited range of motion (ROM) of the lower extremities has long been investigated as an associated factor for LBP in juvenile athletes [11, 12]. Although one prospective cohort study revealed that the limited straight-leg-raising (SLR) angle might be a risk factor for LBP in high school baseball players [11], another prospective study reported that lower extremity muscle extensibility was not associated with the risk of LBP among young basketball and floorball players [12]. The discrepancies in the results from different studies might be influenced by sport type, sex, training frequency, and age. Furthermore, the association between aging and decreased ROM of the lower extremities during school age due to a growth spurt is well established [13]. A study investigating age-related differences in the ROM in juvenile soccer players showed a slight tendency to reduce the hip ROM and to increase the SLR angle for different ages [14]. Furthermore, the ROM of the upper extremities of juvenile baseball players was reduced as age increased and was related to shoulder pain during throwing motions [15]. Therefore, participation in sports activities and aging might influence the ROM of the extremities, which might be associated with musculoskeletal problems. Although several cross-sectional and prospective studies have investigated the ROM of the lower extremities and LBP in high-school baseball players [5, 11], the wide range of age-related differences in the ROM of the lower extremities and their relationship with LBP remains unclear. The ROM of the lower extremities and LBP may interact with each other in young baseball players.

The purpose of this study was to investigate age-related differences in the ROM of the lower extremities and their relationship to LBP among school-aged baseball players aged 6–16 years using a cross-sectional design. We hypothesized that the ROM of the lower extremities would significantly decrease with age and would be positively correlated with LBP among school-aged baseball players.

## Methods

### Study Design

This cross-sectional study involved baseball teams among all local communities in the Fukushima Prefecture of Japan. The study was conducted according to the Declaration of Helsinki. The Research Ethics Committee of Fukushima Medical University approved our cross-sectional study protocol (identification numbers 2063, 2064). All the parents/guardians and participants provided written informed consent or assent before enrollment.

### Participants

A total of 7737 baseball players (1730 elementary, 3236 junior high school, and 2771 high-school students) were registered in 2018 in the Fukushima Prefecture of Japan. Medical checkups were conducted for elementary or junior high school baseball players who regularly attended the local baseball competitions in the Fukushima Prefecture of Japan from 2018 to 2019. Further, the medical checkups for elementary or junior high school baseball players were conducted each year between October and December. In addition, annual medical checkups for high school baseball players among the entire local communities in the Fukushima Prefecture were conducted from 2018 to 2019. The medical checkups for high school baseball players were conducted immediately after the end of the annual sports season (between October and December each year). The eligibility criterion for this study was “school students who participated in medical checkups”. If the same student participated in medical checkups in multiple years, only data from the most recent year were adopted.

### Characteristics of the Participants

Medical checkups comprised two items: a self-completed questionnaire and physical examination. The self-completed questionnaires were distributed to the participants through the postal service before the medical checkup and collected on the checkup day. The questionnaire for elementary students was developed specifically for this study in Japanese writing hiragana letter forms, which are easy to understand by the younger participants. If the younger participants needed help answering the questionnaires, the questionnaire was completed by a parent/guardian. The questionnaire items included sex, age, playing position, years of baseball experience, and total amount of practice time per week (h). Any musculoskeletal injury in the hip/groin, thigh, and knee presented during the 1 year before the study was also evaluated. The essential data, including those on presenting symptoms at onset and at the time of the study, number of hospital visits, definite diagnosis, and results of investigations, were recorded. Players who had practiced and played as pitchers were considered pitchers, even if they also played other positions. Anthropometric measurements were taken on the day of the checkup. Height was measured to the nearest 0.1 cm using a portable anthropometer. Weight was measured to the nearest 0.1 kg on portable scales. The Rohrer Index (calculated as weight (kg)/height (cm)^3^ *10^7^) was assessed to evaluate the degree of obesity in the participants [16]. In Japan, the Rohrer index is widely used as an indicator to represent the physique of children, including elementary and junior high school students. Accordingly, individuals with a Rohrer index of > 145 are considered to be overweight in Japan [17].

### ROM of the Lower Extremities

The ROM of the lower extremities was assessed using standard physical examination techniques. The ROM of the lower extremities was measured on the day of the medical checkup between 9 and 12 am, and the Thomas test, SLR test, and heel-to-buttock-test (HBT) were performed. All participants wore baseball practice uniforms with long-sleeved shirts and long trousers. The venue of the medical checkup was an indoor conference room or a gymnasium. All lower extremity measures were obtained bilaterally. For the Thomas test, the participants were positioned supine on the examination table, and the examiner passively flexed one hip, bringing the knee up to the chest to flatten the lumbar spine and to stabilize the pelvis. Test results were defined as positive if, following the flexion of the opposite hip, the knee lifted off the examination table [11]. For the SLR angle measurement, the player was asked to lie supine on an examination table and the leg passively elevated with the hip and knee fully extended, and a goniometer then determined the SLR angle. The SLR angle was defined as limited if the elevation angle was < 70° [5, 11, 18]. To accurately measure this ROM, the testing procedure in this study provided suitable stabilization of the pelvis during the SLR test. In addition, the ankle was relaxed throughout the test to minimize the influence of the gastrocnemius muscle in this study [19, 20]. The HBT was performed in the prone position, and the knee was passively flexed; moreover, it was defined as positive if the heel did not touch the buttock [21]. Fifteen well-trained board-certified physiotherapists performed all procedures. Examiners who performed each assessment (the Thomas test, SLR test, and HBT) were blinded to the status of LBP and results of other physical examinations. To minimize the measurement error, all physical examinations were standardized, and all physiotherapists attended two workshops before the study was conducted. In addition, the first or second author, both board-certified orthopedic surgeons, supervised the standardized physical examinations in both the workshops and day of the medical checkup.

### Evaluation of LBP

Previous and seasonal LBP episodes during the preceding year were measured by the self-completed questionnaire. Previous episodes of LBP were assessed using the following question: “Have you ever felt pain in your low back?” (0 = “no”; 1 = “yes”). Episodes during the last year of LBP were assessed using the following question: “Have you felt pain in your lower back within the previous year?” (0 = “not at all”; 1 = “I felt low back pain and rested from practice for < 1 week”; 2 = “I felt low back pain and rested from practice for 1–4 weeks; 3 = “I felt low back pain and rested from practice for > 4 weeks”). LBP during the season was defined as any answers from 1 to 3. To assess the characteristics of present LBP, pain during lumbar flexion or extension (spinal sign) on the day of the medical checkup was also evaluated [5]. Participants were asked to stand in a relaxed position with their feet shoulder-width apart. From this position, they were asked to perform maximal flexion of the lumbar spine, followed by maximum extension of the lumbar spine with the legs straight. LBP was recorded as positive if a participant complained of pain localized between the costal margins and superior gluteal folds during that test. The examiners who performed the assessments of the spinal sign on the day of the medical checkup were blinded to the results of the assessments of the ROM of the lower extremities.

### Statistical Analyses

Participants with complete data were included in the primary analysis. Descriptive statistics were calculated for the participants’ characteristics. Continuous data were summarized as medians and interquartile range, while dichotomous or categorical data were summarized as proportions. The prevalence of seasonal LBP and limited ROM of the lower extremities at each age were investigated bilaterally. Chi-square or Fisher exact test was used to investigate the relationship between seasonal LBP and the limited ROM of the lower extremities for each side stratified by age groups (< 10 years, 11–12 years, 13–14 years, and 15–16 years). A multivariable logistic regression analysis was performed to investigate the association between the limited ROM of the lower extremities and LBP. We restricted the age group for the model based on the results of univariate analyses (*P* < 0.05), and the following were analyzed as explanatory variables: total amount of practice per week greater than the median of this study population, Rohrer index of > 145, any previous history of lower extremity injuries during the season, and limited ROM of the lower extremities on either side. Odds ratios (ORs) and 95% confidence intervals (CIs) were calculated. All statistical analyses were conducted using JMP version 15.0.0 software (SAS Institute, Cary, NC, United States). All tests used were two-sided, and statistical significance was set at *P* < 0.05. The protocol and analysis plan of this study were not pre-registered, and therefore, all results of this study should be considered exploratory.

## Results

**Fig. 1:**
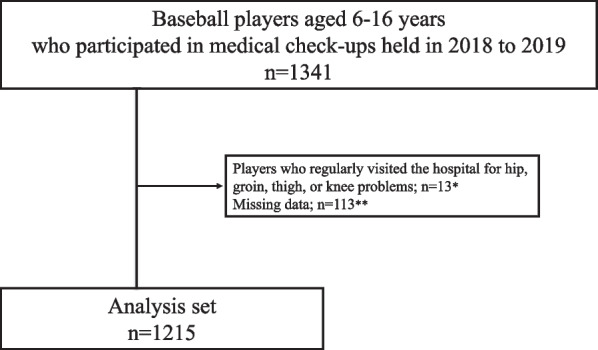
Study flowchart

A total of 1341 players from 129 (47 elementary school, 39 junior high school, and 43 high school) baseball teams initially participated in the medical checkups. Thirteen players who regularly visited hospitals for lower extremity problems and 113 for whom data were missing were excluded (Additional file 1: Table S1 and Additional file 2: Table S2). As a result, 1215 players (216 pitchers, 999 fielders) were included in this study (Fig. 1).

The players excluded with missing data were significantly younger than those included in this study (Additional file 3: Table S3). However, the excluded players were not significantly different from those who were included in terms of the prevalence of seasonal LBP and limited ROM of the lower extremities in all age groups (Additional file 3: Table S3). A summary of the participants’ characteristics is presented in Table 1.

**Table 1:** Summary of the demographic data

Characteristics	Total n = 1215	Age group by years			
		≤ 10 n = 195	11–12 n = 290	13–14 n = 320	15–16 n = 410
Position					
Pitcher, n (%)	216 (17.8)	13 (6.7)	75 (25.9)	43 (13.4)	85 (20.7)
Fielder, n (%)	999 (82.2)	182 (93.3)	215 (74.1)	277 (86.6)	325 (79.3)
Gender					
Male, n (%)	1193 (98.2)	183 (93.8)	283 (97.6)	317 (99.1)	410 (100)
Height					
Median (IQR), cm	163 (147–170)	136 (132–142)	146 (141–154)	164 (159–168)	170 (167–174)
Body mass					
Median (IQR), kg	54 (41–64)	32 (29–38)	41 (35–49)	54 (48–60)	65 (59–71)
Rohrer index					
Median (IQR)	127 (117–139)	128 (118–144)	127 (117–140)	122 (112–134)	131 (122–141)
Years of baseball experience					
Median (IQR), years	4 (3–6)	2 (1–4)	3 (2–5)	4 (2–5)	7 (5–8)
Total amount of practice per week					
Median (IQR), h	15 (11–20)	12 (10–16)	12 (10–16)	12 (10–15)	24 (20–30)
Previous history of musculoskeletal injuries during the season					
Hip/Groin, n (%)	13 (1.1)	0 (0.0)	0 (0.0)	4 (1.3)	9 (2.2)
Thigh, n (%)	14 (1.2)	0 (0.0)	1 (0.3)	4 (1.3)	9 (2.2)
Knee, n (%)	36 (3.0)	0 (0.0)	5 (1.7)	9 (2.8)	22 (5.4)
Positive Thomas test					
TS, n (%)	515 (42.4)	61 (31.3)	120 (41.4)	134 (41.9)	200 (48.8)
NTS, n (%)	498 (41.0)	54 (27.7)	108 (37.2)	133 (41.6)	203 (49.5)
Positive SLR test					
TS, n (%)	262 (21.6)	25 (12.8)	59 (20.3)	80 (25.0)	98 (23.9)
NTS, n (%)	229 (18.8)	24 (12.3)	57 (19.7)	77 (24.1)	71 (17.3)
Positive HKT					
TS, n (%)	517 (42.6)	19 (9.7)	91 (31.4)	159 (49.7)	248 (60.5)
NTS, n (%)	527 (43.4)	20 (10.3)	93 (32.1)	166 (51.9)	248 (60.5)

Data are presented as medians and interquartile ranges (IQRs) or numbers and percentages

*TS* throwing arm side, *NTS* non-throwing arm side, *SLR* straight leg raising, *HKT* heel-to-buttock test

**Fig. 2:**
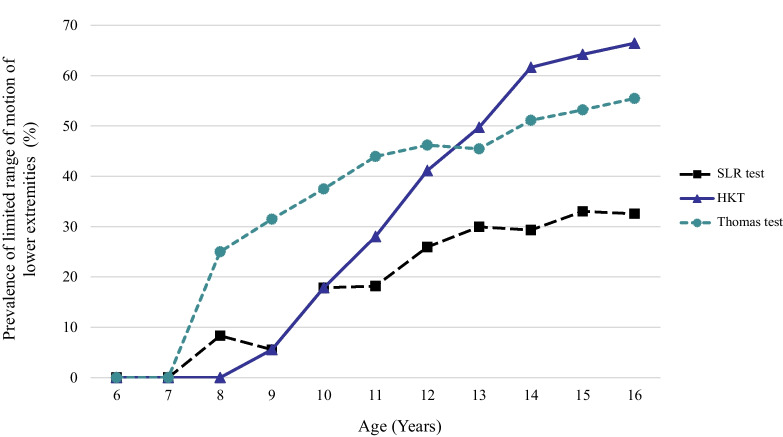
Prevalence of limited range of motion of lower extremity by age

Of the 1215 participants, 1193 (98.2%) were male individuals. They had a median (interquartile range) of 4 (3–6) years of baseball experience, and the median (interquartile range) total amount of practice time per week was 15 (11–20) h. The prevalence of a positive Thomas test, SLR test, and HBT all increased with age (Fig. 2). Among the tests, the prevalence of a positive Thomas test increased early, at 8 years of age, followed by an increase in a positive SLR test and HBT from 10 years of age. The increasing prevalence rate of limited ROM was higher for the HBT (17.9% at 10 years of age to 66.5% at 16 years of age) than for the SLR test (17.9% at 10 years of age to 32.6% at 16 years of age) and Thomas test (37.5% at 10 years of age to 55.5% at 16 years of age).

**Fig. 3:**
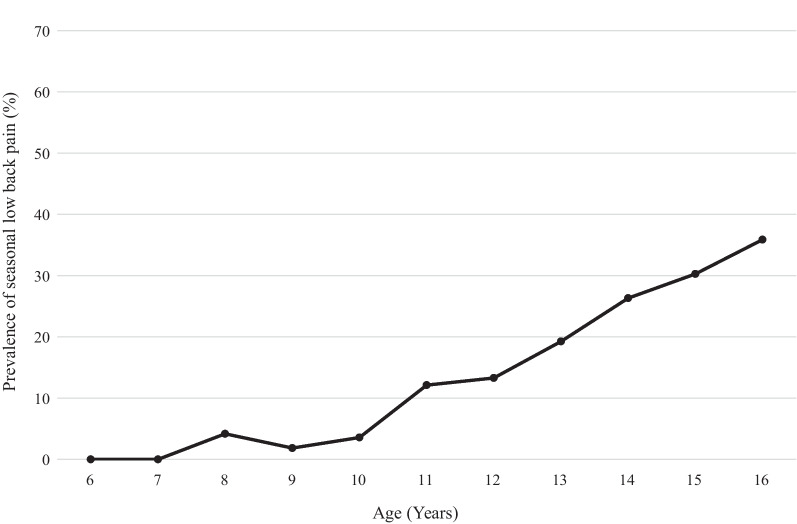
Prevalence of seasonal low back pain by age

Of the 1215 players, 255 (21.0%) reported seasonal LBP requiring rest during the previous year (Table 2), and the prevalence of seasonal LBP with a rest period increased with age (Fig. 3).

**Table 2:** Characteristics of low back pain

Low back pain	Total n = 1215	Age group by years			
		≤ 10 n = 195	11–12 n = 290	13–14 n = 320	15–16 n = 410
Previous episodes, n (%)	305 (25.1)	7 (3.6)	39 (13.5)	84 (26.3)	175 (42.7)
Seasonal pain with required rest, n (%)					
Total	255 (21.0)	6 (3.1)	37 (12.8)	71 (22.2)	142 (34.6)
Grade 1 (< 1 week of rest)	192 (15.8)	5 (2.6)	33 (11.4)	49 (15.3)	105 (25.6)
Grade 2 (1–4 weeks of rest)	46 (3.8)	1 (0.5)	3 (1.0)	18 (5.6)	24 (5.9)
Grade 3 (≥ 4 weeks of rest)	17 (1.4)	0 (0.0)	1 (0.3)	4 (1.3)	12 (2.9)
Low back pain on day of checkup, n (%)	146 (12.0)	10 (5.1)	18 (6.2)	41 (12.8)	77 (18.8)
Pain at lumbar flexion	38 (3.1)	4 (2.1)	3 (1.0)	11 (3.4)	20 (4.9)
Pain at lumbar extension	136 (11.2)	8 (4.1)	17 (5.9)	36 (11.3)	75 (18.3)

Data are presented as numbers and percentages. The data represent the comparison between a pitcher and fielder

A positive HBT in both the throwing and non-throwing arm sides (*P* = 0.0051 and *P* = 0.021, respectively) was associated with seasonal LBP in the 11–12 year age group (Table 3). Additionally, a positive Thomas test in the throwing arm side (*P* = 0.048) was associated with seasonal LBP in the 13–14 year age group in the univariate analysis (Table 3).

**Table 3:** Univariate analysis for the association between LBP and limited range of motion of the lower extremities by age categories

Age ≤ 10 years, n = 195	Players without seasonal LBP n = 960	Players with seasonal LBP n = 255	*P* value
	n = 189	n = 6	
Positive Thomas test			
TS	59 (31.2)	2 (33.3)	1.00
NTS	52 (27.5)	2 (33.3)	0.67
Positive SLR test			
TS	25 (13.2)	0 (0.0)	0.34
NTS	23 (12.2)	1 (16.7)	0.74
Positive HKT			
TS	19 (10.1)	0 (0.0)	0.41
NTS	20 (10.6)	0 (0.0)	0.40

Age 11–12 years, n = 290	n = 253	n = 37	
Positive Thomas test			
TS	103 (40.7)	17 (46.0)	0.55
NTS	90 (35.6)	18 (48.7)	0.12
Positive SLR test			
TS	47 (18.6)	12 (32.4)	0.06
NTS	46 (18.2)	11 (29.7)	0.10
Positive HKT			
TS	72 (28.5)	19 (51.4)	0.0051*
NTS	75 (29.6)	18 (48.7)	0.021*

Age 13–14 years, n = 320	n = 249	n = 71	
Positive Thomas test			
TS	97 (39.0)	37 (52.1)	0.048*
NTS	102 (41.0)	31 (43.7)	0.68
Positive SLR test			
TS	65 (26.1)	15 (21.1)	0.39
NTS	60 (24.1)	17 (23.9)	0.98
Positive HKT			
TS	118 (47.4)	41 (57.8)	0.12
NTS	124 (49.8)	42 (59.2)	0.16

Age 15–16 years, n = 410	n = 269	n = 141	
Positive Thomas test			
TS	128 (47.6)	72 (51.1)	0.50
NTS	131 (48.7)	72 (51.1)	0.65
Positive SLR test			
TS	66 (24.5)	32 (22.7)	0.68
NTS	49 (18.2)	22 (15.6)	0.51
Positive HKT			
TS	160 (59.5)	88 (62.4)	0.56
NTS	159 (59.1)	89 (63.1)	0.43

*LBP* low back pain, *TS* throwing arm side, *NTS* non-throwing arm side, *SLR* straight leg raising, *HKT* heel-to-buttock test

^*^P < 0.05

After adjusting for factors associated with LBP using logistic regression modeling restricted by players aged 11–14 years, significant associations between the positive HBT (OR 1.75, 95% CI 1.11–2.78; *P* = 0.016), the total amount of practice per week ≥ 20 h (OR 2.92, 95% CI 1.59–5.27; *P* = 0.0007), a Rohrer index of ≥ 145 (OR 2.12, 95% CI 1.24–3.59; *P* = 0.0069), and LBP were found (Table 4).

**Table 4:** Multivariable analysis for the association between LBP and limited range of motion of the lower extremities (11–14 year age group)

	OR (95% CI)	*P* value
Total amount of practice per week, ≥ 20 h	2.92 (1.59–5.27)	0.0007*
Rohrer index, ≥ 145	2.12 (1.24–3.59)	0.0069*
Previous history of lower extremity injuries during the season	1.61 (0.51–4.30)	0.39
Positive Thomas test	1.24 (0.79–1.93)	0.34
Positive SLR test	0.99 (0.59–1.64)	0.98
Positive HKT	1.75 (1.11–2.78)	0.016*

*LBP* low back pain, *OR* odds ratio, *CI* confidence interval, *SLR* straight leg raising, *HKT* heel-to-buttock test

^*^P < 0.05

## Discussion

Our study revealed that the prevalence of LBP and limited ROM of the lower extremities increased with advancing age from 6 to 16 years and that a positive HBT was associated with LBP in baseball players aged 11–14 years.

The relationship between the ROM of the lower extremities and LBP occurrence among juvenile athletes has long been debated. One cross-sectional study that evaluated 130 athletic patients (aged 8–17 years) with LBP indicated that the limitation of both the SLR angle and heel-to-buttock-distance was significantly related to lumbar stress fractures [22]. Similarly, a prospective cohort study of 335 baseball players (aged 15–16 years) found that a limited SLR angle was a risk factor for LBP development [11]. On the contrary, a prospective 1-year follow-up cohort study of 86 adolescent athletes (aged 10.3–13.3 years) reported no association between limited SLR and hip flexor muscle tightness and LBP occurrence [23], and a cross-cross sectional study also found no association between hamstring tightness and severe LBP [24].

The relationship between aging and lower extremity ROM reduction in both athletic and non-athletic populations has been reported in previous studies [13, 25]. Our data demonstrated an increasing trend in the limited ROM of the lower extremities with the increasing age of juvenile baseball players. In particular, the most dramatic increase in the limited ROM prevalence was found in the HKT group from ages 10 to 14 years, with an almost threefold increase. The HKT is commonly used to measure the ROM of the knee joint and tightness of the quadriceps femoris muscle [11, 21]. We excluded participants who were regularly referred to the hospital for lower extremity problems in this study; a positive HKT might have been influenced by the tightness of the quadriceps femoris muscle. Muscle tightness of the quadriceps femoris muscle in juvenile athletes has been reported as a potential risk factor for musculoskeletal pain, which refers to pain in areas such as the knee, elbow, and shoulder [26, 27]. Cross-sectional studies targeting young baseball players (aged 7–14 years) and football players (aged 10–15 years) revealed that the degree of quadriceps tightness increased with age as the skeletal maturation of the tibial tuberosity advanced, while the degree of hamstring tightness stayed unchanged or decreased [27, 28]. According to the findings of these studies and our results, the tightness of the quadriceps femoris muscle might have a greater impact on the musculoskeletal system than that of the hamstring muscle in juvenile athletes.

Regarding LBP, the tightness of the quadriceps femoris muscle might generate an increased force on the posterior element of the lumbar spine by restricting the posterior tilt of the pelvis and producing excessive lumbar lordosis. The causes of LBP in school-aged children are broad and differ from those observed in adulthood [29]. One of the most common causes of LBP in school-aged children might be acute or subacute mechanical LBP, which is used synonymously with the terms posterior overuse syndrome or hyperlordotic LBP [29, 30]. Lumbar spondylolysis’ incidence is reportedly much higher in young athletes, especially baseball players [7, 30]. The tightness of the quadriceps femoris muscle arising from rapid growth might increase lumbar lordosis and place excessive mechanical stress on the pars interarticularis, subsequently leading to LBP due to lumbar spondylolysis. In addition, such kinetic alterations caused by limited ROM of the lower extremity could induce an inadequate energy transfer from the lower limb to the upper limb and might trigger compensatory movements and undue stress on the lumbar spine and pelvis in baseball players [31]. On the other hand, we were unable to assess the direction of causality for the relationship between the tightness of the quadriceps femoris muscle and LBP in this cross-sectional study. LBP might induce the limitation of ROM of the lower extremities due to limited activities.

Our study also revealed that a high amount of practice (≥ 20 h per week) and being overweight (Rohrer index ≥ 145) were factors associated with LBP in baseball players aged 11–14 years. Considering that these variables could affect both muscle tone and LBP, it is important to put them into the multivariate analysis. Previous research has shown a dose–response association between weekly hours of sports participation and sports-related overuse injury, including LBP in childhood and adolescence [32–34]. Moreover, LBP during the past 12 months was associated with the amount of training during that time in adolescent athletes (10.3–13.3 years of age) [35]. Our results are in line with those of another study indicating that athletes aged 7–18 years who participate in more weekly hours of sport than their age have an increased risk of sustaining a sports-related injury (odds ratio 1.59, 95% CI 1.17–2.16) [32].

Weight gain has also been reported as a risk factor for LBP in the younger age group [36]. Nevertheless, despite the frequent reports of the association between obesity and LBP, there might not be a clear causal relationship between them [37]. Further, the relationship between obesity and LBP may contribute to a vicious cycle in which obesity, LBP, and poor fitness reinforce one another. Moreover, being overweight could be the factor associated with not only LBP but also elbow injuries [38, 39], as well as with high amounts of practice. Therefore, these factors might be deserved attention in school-aged baseball players.

Some limitations of this study should be acknowledged. First, this study relied on the self-reports of the participants aged 6–16 years; thus, recall bias and variable parental involvement might have occurred. Consequently, some data that were collected by our author-developed questionnaire, such as the total amount of practice time per week, might not have been precise. Second, considering the number of baseball players, we only recruited those who regularly attended the local baseball competitions registered in 2018 in our prefecture of Japan. Therefore, selection bias could be imposed by selectively recruiting competitive players rather than recreational players. Third, despite our interpretation of a positive HKT being indicative of quadriceps femoris muscle tightness in the discussion, it is possible that the measured values were affected by muscle tone in the gluteal area and ROM in the knee joint. To mitigate this potential issue, we excluded participants who regularly sought medical treatment for lower extremity problems and incorporated their previous history of lower extremity injuries during the season as an explanatory variable in our multivariable analysis. Fourth, we did not include the evaluation of the mobility of the whole spine, including the lumbar spine, in this study. The spine's mobility that influences both the ROM of the lower extremities and LBP might be a positive confounder, and our results might overestimate the association between the two. Fifth, imaging analyses of the participants were lacking; therefore, the causes of LBP pathology in this study were uncertain. Sixth, this study only included a small number of female players; therefore, the results may not be generalizable to female athletes. Finally, because this was a cross-sectional study, we were unable to assess the direction of causality for the relationship between the positive HKT and LBP. Prospective longitudinal studies are required to confirm these relationships.

## Conclusions

A positive HKT might be potentially associated with LBP occurrence in baseball players aged 11–14 years. Regarding clinical relevance, a particular emphasis on the limited ROM of the knee joint and tightness of the quadriceps femoris muscle might be evaluated for school-aged baseball players.

## Supplementary Information

**Additional file 1: Table S1.** The detailed diagnosis in players excluded from the study due to regular hospital visits for lower extremity problems.

**Additional file 2: Table S2.** Details of missing data.

**Additional file 3: Table S3.** Comparison between participants with complete data and those with missing variables.

## Data Availability

The datasets used and analyzed during the current study are available from the corresponding author upon reasonable request.

## Abbreviations

CI: Confidence interval
HBT: Heel-to-buttock-test
LBP: Low back pain
NTS: Non-throwing arm side
OR: Odds ratio
SD: Standard deviation
SLR: Straight-leg-raising
TS: Throwing arm side
