# Supplementary material for: Age-Related Differences in the Limited Range of Motion of the Lower Extremity and Their Relation to Low Back Pain in Young Baseball Players: A Cross-Sectional Study of 1215 Players
Source: Sports Med Open. 2023 May 3;9:26. doi: 10.1186/s40798-023-00572-w (PMC10156885; doi:10.1186/s40798-023-00572-w)
Supplement: Supplementary file 1 — Additional file 1: Table S1. The detailed diagnosis in players excluded from the study due to regular hospital visits for lower extremity problems. [file 40798_2023_572_MOESM1_ESM.docx]

Supplemental Table 1. The detailed diagnosis in players excluded from the study due to regular hospital visits for lower extremity problems

| Diagnosis | Number |
| --- | --- |
| Hip/Groin (n=6) |  |
| Groin pain syndrome | 3 |
| Stress fracture of pubic ramus | 1 |
| Avulsion fracture of anterior superior iliac spine | 1 |
| Acetabular dysplasia | 1 |
| Thigh (n=2) |  |
| Hamstrings injury | 1 |
| Quadriceps injury | 1 |
| Knee (n=5) |  |
| Osteochondritis dissecans | 2 |
| Anterior cruciate ligament injury | 1 |
| Osgood-Schlatter disease | 1 |
| Unknown | 1 |
| Total | 13 |
|  |  |
