# Supplementary material for: Age-Related Differences in the Limited Range of Motion of the Lower Extremity and Their Relation to Low Back Pain in Young Baseball Players: A Cross-Sectional Study of 1215 Players
Source: Sports Med Open. 2023 May 3;9:26. doi: 10.1186/s40798-023-00572-w (PMC10156885; doi:10.1186/s40798-023-00572-w)
Supplement: Supplementary file 2 — Additional file 2: Table S2. Details of missing data. [file 40798_2023_572_MOESM2_ESM.docx]

Supplemental Table 2. Details of missing data

| Variables | number |
| --- | --- |
| Height and weight | 80 |
| Total amount of practice time | 20 |
| Physical findings | 13 |
| Years of baseball experience | 3 |
| Total | 113* |
| *Three subjects had duplicate missing values (two players with missing height and weight and physical findings and one with missing height and weight and years of baseball experience) |  |
