# Supplementary material for: Age-Related Differences in the Limited Range of Motion of the Lower Extremity and Their Relation to Low Back Pain in Young Baseball Players: A Cross-Sectional Study of 1215 Players
Source: Sports Med Open. 2023 May 3;9:26. doi: 10.1186/s40798-023-00572-w (PMC10156885; doi:10.1186/s40798-023-00572-w)
Supplement: Supplementary file 3 — Additional file 3: Table S3. Comparison between participants with complete data and those with missing variables. [file 40798_2023_572_MOESM3_ESM.docx]

Supplemental Table 3. Comparison between participants with complete data and those with missing variables

|  |  | Players with complete data  n=1228 | Players with missing values  n=113 | P-value |
| --- | --- | --- | --- | --- |
| Age, in years | | | | |
| ≤10, n=244 |  | 196 (16.0) | 48 (42.5) | <0.0001* |
| 11-12, n=332 |  | 290 (23.6) | 42 (37.2) |  |
| 13-14, n=339 |  | 323 (26.3) | 16 (14.2) |  |
| 15-16, n=426 |  | 419 (34.1) | 7 (6.2) |  |
| LBP and limited range of motion of the lower extremities | | | | |
| Age ≤10, n=244 |  | n=196 | n=48 |  |
| Seasonal low back pain, n (%) |  | 6/196 (3.1) | 1/48 (2.1) | 0.72 |
| Positive Thomas test | TS | 62/196 (31.6) | 8/38 (21.1) | 0.19 |
|  | NTS | 54/196 (27.6) | 6/38 (15.8) | 0.13 |
| Positive SLR test | TS | 25/196 (12.8) | 7/46 (15.2) | 0.66 |
|  | NTS | 24/196 (12.2) | 5/46 (10.9) | 0.80 |
| Positive HKT | TS | 20/196 (10.2) | 4/46 (8.7) | 0.76 |
|  | NTS | 20/196 (10.2) | 3/46 (6.5) | 0.59 |
| Age 11-12, n=332 |  | n=290 | n=42 |  |
| Seasonal low back pain, n (%) | n (%) | 37/290 (12.8) | 4/42 (9.5) | 0.55 |
| Positive Thomas test | TS | 120/290 (41.4) | 13/38 (34.2) | 0.40 |
|  | NTS | 108/290 (37.2) | 10/38 (26.3) | 0.19 |
| Positive SLR test | TS | 59/290 (20.3) | 7/38 (18.4) | 0.78 |
|  | NTS | 57 / 290 (19.7) | 9/38 (23.7) | 0.56 |
| Positive HKT | TS | 91/290 (31.4) | 22/38 (57.9) | 0.0012* |
|  | NTS | 93/290 (32.1) | 23/38 (60.5) | 0.0006* |
| Age 13-14, n=339 |  | n=323 | n=16 |  |
| Seasonal low back pain, n (%) | n (%) | 72/323 (22.3) | 4/16 (25.0) | 0.80 |
| Positive Thomas test | TS | 13/323 (42.4) | 10/16 (62.5) | 0.11 |
|  | NTS | 136/323 (42.1) | 9/15 (60.0) | 0.17 |
| Positive SLR test | TS | 80/323 (24.8) | 7/15 (46.7) | 0.06 |
|  | NTS | 77/323 (23.8) | 7/16 (43.8) | 0.07 |
| Positive HKT | TS | 160/323 (49.5) | 9/16 (56.3) | 0.60 |
|  | NTS | 167/323 (51.7) | 8/16 (50.0) | 0.89 |
| Age 15-16, n=426 |  | n=419 | n=7 |  |
| Seasonal low back pain, n (%) |  | 142/419 (33.9) | 0/7 (0.0) | 0.10 |
| Positive Thomas test (either side) | TS | 207/419 (49.4) | 1/7 (14.3) | 0.12 |
|  | NTS | 210/419 (50.1) | 1/7 (14.3) | 0.12 |
| Positive SLR test (either side) | TS | 101/419 (24.1) | 1/7 (14.3) | 1.00 |
|  | NTS | 73/419 (17.4) | 1/7 (14.3) | 1.00 |
| Positive HKT (either side) | TS | 254/419 (60.6) | 4/6 (66.7) | 1.00 |
|  | NTS | 256/419 (61.1) | 4/7 (57.1) | 1.00 |

* P<0.05; Chi-square tests were used for the <10, 11-12, and 13-14 year age groups. Fisher exact tests were used for the 15-16 year age group.
